# Factors associated with patient satisfaction towards pharmacy services among out-patients attending public health clinics: Questionnaire development and its application

**DOI:** 10.1371/journal.pone.0241082

**Published:** 2020-11-10

**Authors:** Aniza Ismail, Yan Nee Gan, Norfazilah Ahmad

**Affiliations:** 1 Department of Community Health, Faculty of Medicine, Universiti Kebangsaan Malaysia, Cheras, Kuala Lumpur, Malaysia; 2 Malaysian Health Technology Assessment Section, Ministry of Health Malaysia, Federal Government Administrative Centre, Putrajaya, Malaysia; University of South Australia, AUSTRALIA

## Abstract

**Introduction:**

Patient satisfaction is widely used to measure quality of healthcare by identifying potential areas for improvement. Aim of study is to assess patient satisfaction towards pharmacy services and its associated factors using newly developed questionnaire among outpatients attending public health clinics.

**Materials and methods:**

Public Health Clinic Patient Satisfaction Questionnaire (PHC-PSQ) towards pharmacy services was developed using exploratory factor analysis and Cronbach’s α. A cross-sectional study was conducted among 400 patients visiting the pharmacy in three randomly selected public health clinics recruited via systematic random sampling. Data was collected using a set of questionnaire including PHC-PSQ. Factors associated with patient satisfaction was analysed using multiple linear regression.

**Results:**

Final PHC-PSQ consisted of three domains (administrative competency, technical competency and convenience of location) and 22 items with 69.9% total variance explained. Cronbach's α for total items was 0.96. Total mean score for patient satisfaction was 7.56 (SD 1.32). Older age and higher education were associated with lower patient satisfaction mean score. Patients who had visited the pharmacy more than once in the past three months, perceived to be in better health status and had a more correct general knowledge of pharmacists expressed higher patient satisfaction mean score.

**Conclusions:**

PHC-PSQ is a newly developedtool to measure patient satisfaction towards pharmacy services in public health clinics in Malaysia. Patient satisfaction was relatively high. Age, education, frequency of visit, self-perceived health status and general knowledge of pharmacists were factors significantly associated with patient satisfaction.

## Introduction

The role of pharmacists in public out-patient health clinics in Malaysia has broadened from traditional dispensing services to include patient-centred pharmaceutical care services. Multidisciplinary approach in public health clinics allows pharmacists to work closely with other healthcare workers (HCWs) (e.g. doctors, nurses and medical assistants) to optimize patient-centred healthcare services.

Patient satisfaction is used to measure the quality of healthcare, identifying potential areas for improvement to increase the effectiveness of healthcare system [1, 2]. A local study conducted among patients visiting public health clinics showed that the more satisfied groups were less educated, older and of Indian and Chinese ethnic groups [3]. As pharmacy services in the public health clinics expand throughout the years, an update on factors associated with patient satisfaction was warranted.

Several questionnaires have been developed to measure patient satisfaction towards pharmacy services such as Pharmacy Services Questionnaire (PSQ) [4], Pharmacist Services Questionnaire (PSPSQ) [5], and Pharmaceutical Care Satisfaction Questionnaire (PCSQ) [6]. Among these questionnaires, the PSPSQ, consisting of 16 items divided into two domains (quality of care and interpersonal relationship), was translated and validated in Malay language [7]. Validation study was conducted among patients who had encountered a pharmacist at any public or private hospital. Another questionnaire was also developed and validated in an out-patients centre situated in a tertiary hospital in Malaysia [8]. To date, no questionnaire has been developed specifically to assess patient satisfaction for public health clinic pharmacy services in Malaysia. The setting of these clinics differs from private sectors and tertiary hospitals. The aim of this study was to assess the patientsatisfaction towards pharmacy services and its associated factors using newly developed questionnaire among out-patients attending public health clinics.

## Materials and methods

### Study setting and population

Approval was obtained from Universiti Kebangsaan Malaysia Medical Centre Research Ethics Committee (research code: FF-2015-011) and National Institutes of Health of Ministry of Health Malaysia [research code: NMRR-14-1557-23219 (IIR)]. Written consent was obtained from all patients. Kuala Lumpur is the capital of Malaysia, with an estimated population of 1.8 million in 2018 [9]. The Kuala Lumpur Federal Territory Health Department manages a total of 14 public health clinics where most city dwellers seek their treatment for chronic and acute illnesses. Patients visiting the pharmacy in these clinics were recruited for this study. Inclusion criteria were patients ≥ 18 years old, Malaysian citizens, registered patients in the clinic, had previously visited the pharmacy in the past three months, and currently on medications for chronic diseases such as diabetes, hypertension and dyslipidaemia for ≥ three months. Exclusion criteria were those who were unable to read the Malay language or experiencing acute symptoms during study and thus deemed unfit to participate.

### Development of Public Health Care Patient Satisfaction Questionnaire (PHC-PSQ)

Evaluation items of patient satisfaction was initially based on satisfaction measures in previous studies [10–12] and modified according to the type of pharmacy services provided in public health clinics. Content validity was conducted by a panel of experts consisting of public health specialists and pharmacists from both government and private sectors. Items were reviewed via professional judgment on relevance to the basic indices of patient satisfaction and appropriateness in terms of simplicity, ambiguity, validity, and sentence structure. PHC-PSQ underwent forward and backward translations. Preliminary version of PHC-PSQ consisted of 36 itemsdivided into five domains: (i) attitude of pharmacists, (ii) provision of special services, (iii) facilities, (iv) convenience of location and (v) convenience of hours. Patients were asked to rate each item based on 10-point scale ranging from 1 (not satisfied) to 10 (very satisfied).

A pre-test was carried out for face validity among five patients who visited the Cheras Health Clinic. A pilot study was then conducted using the preliminary questionnaire among 180 conveniently selected patients who attended the same health clinic. Sample size was determined using subject-to-item ratio of 5:1 [13]. The questionnaire validity was conducted usingexploratory factor analysis (principal components extraction with Varimax rotation). The questionnaire reliability was assessed using Cronbach’s α to assess the internal consistency of all items and each domain. Final revision was made to PHC-PSQ based on statistical analysis and weaknesses identified during pilot study.

### Final study to determine patient satisfaction towards pharmacy services and its associated factor

A cross-sectional study was conducted in three randomly selected public health clinics (Cheras, Jinjang and Tanglin Health Clinics). Patients were recruited using systematic random sampling. A minimum sample size of 328 was calculated based on a previous study [14] using two means formula for continuous outcome [15]. A total of 400 patients was recruited after calculating for possible non-response rate (±20%).

### Data collection

Data were collected between June and August 2015 using a set of self-administered questionnaire consisting of five sections: (A) patients’ socio-demographic characteristics; (B) number of visits to the pharmacy; (C) self-perceived health status; (D) patients' general knowledge of pharmacists; and (E) patient satisfaction towards pharmacy services using the newly developed PHC-PSQ.

Patients’ socio-demographic characteristics included age, gender, race, level of education, employment status as well as self-reported monthly income. The number of visits to the pharmacy was measured by asking respondents to recall the number of visits to the pharmacy in the past three months [4]. Self-perceived health status was measured based on patients' own assessment using a single item rating in five-point scale from 1 (very poor) to 5 (very good) [16, 17]. Patients' general knowledge of pharmacists was measured based on 10 items consisting of seven positive and three negative questions in which a correct answer would contribute to a score of 1, an incorrect answer a score of -1 and unsure answer a score of 0. These items were generated based on World Health Organization (WHO) report describing the qualification and professional roles of community pharmacists [18]. Patient satisfaction was the study outcome measured as continuous variable using the rating scale from 1 to 10. Higher score indicated higher patient satisfaction.

### Statistical analysis

Analyses were performed using SPSS statistics 21.0 software (IBM Corp., Armonk, NY, USA). Categorical data were described as frequency (*n*) and percentage (%). Mean and standard deviation (SD) were used to describe normally distributed data; whereas median and inter-quartile range (IQR) was used to describe non-normally distributed data. Simple linear regression was performed to identify factors associated with patient satisfaction and multiple linear regressionanalysis was performed to control possible confounders. Statistical significance was set at *p* <0.05.

## Results

### Development of the Public Health Care Patient Satisfaction Questionnaire (PHC-PSQ)

Validity analysis from the pilot study indicated that Kaiser-Meyer-Olkin value was 0.93 and Bartlett's test score was χ^2^ = 3134.46 (*p*<0.001). Factor numbers extracted were based on eigenvalues (≥1.0) and scree plot. Three factor numbers were extracted with the total variance explained of 69.9%. A total of 14 items with >5% missing values were omitted. The remaining 22 items had factor loadings >0.40 (Table 1). Reliability analysis showed Cronbach's α for total items was 0.96; first domain, 0.95; second domain, 0.94; and third domain, 0.71 (Table 1). No item was deleted in this analysis. Mean time to complete PHC-PSQ was 7.66 (SD 2.69) minutes. The pilot study yielded the final questionnaire of three domains with 22 items: (i) administrative competency (AC) (9 items), (ii) technical competency (TC) (10 items) and (iii) convenience of location (CoL) (3 items) (Table 1).

**Table 1:** Pilot study: Validity and reliability analyses of the PHC-PSQ.

Dimensions/	Domains	Factor*			ITC	Cronbach’s α
Evaluation items						
		1	2	3		
Directions to the pharmacy are clear.	Administrative competency	0.63			0.76	0.95
The pharmacy is well maintained.		0.73			0.83	
The pharmacy area is clean.		0.81			0.85	
The waiting area is comfortable.		0.83			0.88	
There is sufficient seating in the waiting area.		0.66			0.67	
The pharmacy counter is comfortable.		0.82			0.9	
Operating hours of the pharmacy are satisfactory.		0.66			0.72	
The waiting time to get a queue number at the pharmacy counter is short.		0.76			0.74	
The waiting time to get my medication at the pharmacy counter is short.		0.79			0.79	
The pharmacist is polite and friendly.	Technical competency		0.58		0.72	0.94
The pharmacist provides medications with clear drug label and explanation.			0.62		0.76	
The pharmacist listens to what I have to say.			0.76		0.81	
The pharmacist explains how to take the medications and why it is important to take my medications as directed.			0.78		0.84	
The pharmacist always explains the side effects of medications.			0.8		0.66	
The pharmacist ensures I fully understand the explanation given.			0.74		0.81	
The pharmacist is helpful when I have problems with my medications.			0.8		0.87	
The pharmacist ensures the drugs I need are always in stock.			0.72		0.79	
The pharmacist provides sufficient health-related reading materials such as posters and leaflets.			0.5		0.59	
I feel confident that the drug information provided by the pharmacist is accurate.			0.66		0.8	
There are sufficient parking spaces close to the pharmacy.	Convenience of location			0.79	0.52	0.71
The pharmacy is near public transport.				0.79	0.67	
The pharmacy is near my home.				0.7	0.44	
% of Variance (Total variance explained = 69.9%)		30.6	27.6	11.6		
Cronbach’s α for total items						0.96

ITC, Item-total correlation. Factor 1: administrative competency; Factor 2: technical competency; Factor 3: convenience of location.

*Factor loadings of >0.4.

### Patient satisfaction towards pharmacy services and its associated factor

Table 2 depicts patient characteristics in the final study. The total mean score for patient satisfaction towards overall pharmacy services in government health clinics was high, 7.56 (SD 1.32) (Table 3). Among three domains, highest patientsatisfaction was with AC [mean score 8.47 (SD 1.26)] while the lowest patient satisfaction was with CoL [mean score 5.86 (SD 2.26)]. For AC domain, the item “there is sufficient seating in the waiting area" had the lowest mean score [7.98 (SD 2.07)]. For TC domain, “the pharmacist provides sufficient health-related reading materials such as posters and leaflets” had the lowest mean score [7.51 (SD 2.11)]. All items in the CoL domain had mean score less than the total mean score.

**Table 2:** Characteristics of the patients (*n* = 400).

Characteristics		*n* (%)
Age (years)		
[mean (SD)]: [53.8 (SD 11.4)]		
	18–30	11 (2.8)
	31–40	46 (11.5)
	41–50	83 (20.7)
	51–60	147 (36.8)
	61–70	93 (23.2)
	71–80	20 (5.0)
Gender		
	Male	217 (54.3)
	Female	183 (45.7)
Race		
	Malay	214 (53.5)
	Chinese	106 (26.5)
	Indian	71 (17.8)
	Others	9 (2.2)
Level of education		
	Primary school	53 (13.2)
	Secondary school	207 (51.8)
	College	61 (15.2)
	University	79 (19.8)
Employment status		
	Government servant	76 (19.0)
	Private employee	87 (21.7)
	Self-employed	41 (10.2)
	Retiree	105 (26.3)
	Housewife	62 (15.5)
	Unemployed	26 (6.5)
	Student	3 (0.8)
Income status (Malaysian Ringgit)		
[Median (IQR)]: [1950 (IQR 2800)]		
Frequency of visit to the pharmacy		
	1	130 (32.5)
	2	196 (49.0)
	≥ 3	74 (18.5)
Self-perceived health status		
[mean (SD)]: [3.75 (SD 0.75)]		
Patients’ general knowledge of pharmacists		
[mean (SD)]: [6.12 (SD 2.35)]		

**Table 3:** Mean score for patient satisfaction by each domain and its items.

Domains / Items	Patient satisfaction score
	Mean (SD)
**Domain 1: Administrative competency [Total mean score: 8.47 (SD 1.26)]**	
Directions to the pharmacy are clear.	8.33 (1.69)
The pharmacy is well maintained.	8.59 (1.45)
The pharmacy is clean.	8.85 (1.33)
The waiting area is comfortable.	8.57 (1.60)
There is sufficient seating in the waiting area.	7.98 (2.07)
The pharmacy counter is comfortable.	8.64 (1.49)
Operating hours of the pharmacy are satisfactory.	8.72 (1.36)
The waiting time to get a queue number at the pharmacy counter is short.	8.37 (1.61)
The waiting time to get my medication at the pharmacy counter is short.	8.16 (1.69)
**Domain 2: Technical competency [Total mean score: 8.35 (SD 1.26)]**	
The pharmacist is polite and friendly.	8.65 (1.45)
The pharmacist provides medication with clear drug label and explanation.	8.83 (1.34)
The pharmacist listens to what I have to say.	8.57 (1.40)
The pharmacist explains how to take the medications and why it is important to take my medications as directed.	8.69 (1.39)
The pharmacist always explains the side effects of medications.	7.65 (2.08)
The pharmacist ensures I fully understand the explanation given.	8.31 (1.68)
The pharmacist is helpful when I have problems with my medications.	8.48 (1.47)
The pharmacist ensures the medication I need is always in stock.	8.17 (1.70)
The pharmacist provides sufficient health-related reading materials such as posters and leaflets.	7.51 (2.11)
I feel confident that the drug information provided by the pharmacist is accurate.	8.69 (1.35)
**Domain 3: Convenience of location [Total mean score: 7.56 (SD 1.32)]**	
There are sufficient parking spaces close to the pharmacy.	5.28 (2.77)
The pharmacy is near public transport.	5.68 (2.82)
The pharmacy is near my home.	6.63 (2.65)
**Total items mean score**	**7.56 (1.32)**

Table 4 shows the simple and linear regression analyses. A total of five factors remained significantly associated with patient satisfaction towards pharmacy services in public health clinics (age, number of visits to the pharmacy, level of education, patients’ self-perceived health status and general knowledge of the pharmacists). Interaction analysis between factors showed that older patients (an increase in age by a year) and who perceived themselves in better health status (by a score of one) had higher patient satisfaction mean score by 0.01 [(95% CI 0.00, 0.03), *p* = 0.04]. Patients with high education and who perceived themselves in better health status (by a score of one) had higher patient satisfaction mean score by 0.46 [(95% CI 0.10, 0.82), *p* = 0.01]. With five significant variables and two interaction terms, 13.0% of the variation in mean score for patient satisfaction in the study sample was explained (R^2^ = 0.13). Only part of its application findings had been presented as conference paper at the 9th National Pharmacy R&D Conference in 2016 and the abstract is available at the Malaysian Journal of Pharmacy (https://www.mps.org.my/index.cfm?&menuid=146) [19].

**Table 4:** Factors associated with patient satisfaction towards pharmacy services in public health clinics (*n* = 400).

Factors	Patient satisfaction score	Simple linear regression				Multiple linear regression			
		β	95% CI	*t*	*p*-value	β	95% CI	*T*	*p*-value
	Mean (SD)								
**Age** (years)	7.56 (1.32)	-0.01	-0.02, 0	-2.68	0.01*	-0.01	-0.02, 0	-2.45	0.01*
**Gender**									
Male (reference)	7.47 (1.30)								
Female	7.67 (1.34)	0.20	-0.06, 0.45	1.51	0.13				
**Race**									
Malay (reference)	7.66 (1.34)								
Non-Malay	7.45 (1.28)	-0.21	-0.47, 0.04	-1.61	0.11				
**Level of education**									
Low education (reference)	7.68 (1.25)								
High education	7.34 (1.41)	-0.34	-0.61, -0.07	-2.48	0.01*	-0.51	-0.78, -0.25	-3.80	<0.001*
**Employment status**									
Employed (reference)	7.68 (1.27)								
Unemployed	7.44 (1.36)	0.23	-0.02, 0.49	1.79	0.07				
**Income status**									
≤ MYR2,000 (reference)	7.58 (1.39)								
> MYR2,000	7.54 (1.22)	-0.04	-0.30, 0.21	-0.31	0.76				
**Number of visit to the pharmacy**									
1 (reference)	7.34 (1.33)	0.32	0.05, 0.60	2.32	0.02*	0.26	0, 0.53	2.01	0.04*
> 1	7.67 (1.30)								
**Self-perceived health status**	7.56 (1.32)	0.28	0.11, 0.45	3.23	1.00 x 10–5*	0.24	0.08, 0.41	2.97	3.00 x 10–5*
**Patients' general knowledge of pharmacists**	7.56 (1.32)	0.11	0.06, 0.17	4.25	<0.001*	0.13	0.08, 0.18	4.76	<0.001*
Age*Self-perceived health status						0.01	0, 0.03	2.09	0.04*
Level of education*Self-perceived health status						0.46	0.10, 0.82	2.51	0.01*

*significant at *p*<0.05. Simple linear regression: (Normality and equal variance assumptions for all variables were met and independent random samples were drawn for the construction of data). Multiple linear regression: (adjusted R^2^ = 0.13; The model fits reasonably well; Model assumptions were met; There was no multicollinearity problem; There were significant interaction terms between age and self-perceived health status; level of education and self-perceived health status).

## Discussion

### Development of Public Health Care Patient Satisfaction Questionnaire (PHC-PSQ)

To date, there are no standardized instruments to measure patient satisfaction towards pharmacy services. Each questionnaire was designed to measure different aspect of services as the researchers intended, based on different types of pharmacy services provided and degree of pharmacist-public involvement. Our PHC-PSQ with 22 items, measured the patient satisfaction through three domains (AC, TC and CoL) (S1 Appendix). The recently translated and validated to Malay language (PSPSQ 2.0) among patients visiting the pharmacy at any public or private hospitals had two domains (i.e. quality of care and interpersonal relationship) [7]. The PSPSQ 2.0 did not address patient satisfaction towards facilities and accessibility of services.

Another questionnaire was also recently developed for outpatient pharmacy in a Malaysian public hospital [8]. The Ambulatory Care Pharmacy Services Questionnaire (ACPSQ) has 17 items under five domains (information, accessibility, relationship, outcomes and continuity of care). The ACPSQ addressed the accessibility of services in terms of availability of counselling. Counselling needs may be significantly higher in public hospitals compared to health clinics due to greater severity of diseases being treated and wider range of medications provided. Our study found that many respondents did not understand the terms “dispense” and “counselling” which led to rewording of these phrases, addressed under the TC domain. The patient satisfaction towards facilities was also not addressed in ACPSQ. The Cronbach’s α value for ACPSQ was 0.839 and for each domain ranging from 0.580 to 0.890, which were relatively lower compared to the values obtained in our study.

### Patient satisfaction towards pharmacy services and its associated factor

Our findings showed high level of satisfaction among patients visiting the pharmacy in public health clinics in Malaysia [19]. Similar findings was reported by Lee et al. (2015) with 74.6% of the patients reported to be either “very satisfied” or “satisfied” with the pharmacy services [20]. On the contrary, Soeiro et al. (2017) found that only about half of the patients (58.4%) were overall satisfied with pharmaceutical services in Brazilian primary healthcare [21]. Studies conducted at local public teaching hospitals showed low to moderate levels of patients’ satisfaction on hospital services [22, 23]. These various findings must be interpreted with caution as the patients satisfaction were measured with different definition, measurement tools and type of hospital services.

Our study showed for the AC domain, there was low patient satisfaction mean score for seating facilities in the waiting area [inter-item correlation (ITC): 0.67]. Westaway et al. (2003) also observed that availability of seating in the waiting area (ITC: 0.73) was an important amenity central to the organizational dimension of patient satisfaction, considering the long waiting time in South Africa's public health facilities [24]. For the TC domain, our patients expressed a low score on the provision of health-related reading materials in the pharmacy, which was similar to the finding reported by Oparah et al [12]. Our patients had low patient satisfaction score for all items in CoL domain which is in agreement with previous studies [10–12, 21]. For instance, Soeiro et al. (2017) indicated that 49.5% of the patients were most dissatisfied with the opportunity/convenience domain [21]. The item that was included in their study was also similar with the item in this present study i.e. the proximity of the pharmacy with the patients’ home.

This present study showed that increasing age was significantly associated with lower level of patient satisfaction. This significant finding could be due to two-third of our patients being in the middle age group and older. These older patients are more vulnerable and have higher expectations of care to service their medicine-related needs due to more chronic diseases compared to the younger generation [25]. This previous study also concluded that the expectations of their elderly population on pharmacy services was not met in terms of discussion on the other medicine on their chronic medicine and issues of medicines left from previous visits. We postulated that this unmet expectation could lead to low satisfaction on pharmacy services among these elderly patients. However, our finding contradicts previous studies which showed that older patients were more satisfied with pharmacy services [11, 21]. Kamei et al. (2001) indicated an increase of mean satisfaction score with increased age group [11]. Meanwhile, Soeiro et al. (2017) suggested that patients in the 30–49, 50–69 and ≥70 years age groups had higher odds for aged satisfaction with pharmacy services [odds ratio (OR): 1.22; 95% CI (1.01, 1.47), OR: 1.77; 95% CI (1.39, 2.25) and OR: 2.22; 95% CI (1.63, 3.03), respectively] compared to patients in the 18–29 years age group [21].

Our finding indicates that patients with higher education had lower patient satisfaction score compared to patients with lower education which was congruent with previous studies [3, 16, 26]. For example, a study in Korea which assessed the patient’ satisfaction towards community pharmacies indicated that patients with higher education had lower mean total patients’ satisfaction [3.00 (SD 0.78)] and “friendly expectation” domain [3.23 (SD 0.77)] scores compared to patients with low education [3.32 (SD0.81) and 3.63 (SD 0.77), respectively] [26].

Higher education may be related to greater expectations of healthcare services which may lead to disappointment when expectations are not met [27]. Nevertheless, previous studies found no association between level of education and patient satisfaction [10, 12, 20]. However, majority of study population in two of these studies received up to tertiary education as opposed to one third in our study [10, 12]. As for Lee et al. (2015), college graduate or more was used as reference category versus three other categories (high school graduate, middle school graduate and elementary school graduate or less) in regression analysis which could explain the difference in findings [20].

Higher number of visits to the pharmacy was significantly associated with higher patient satisfaction. This could be due to the fact that patients involved in this study were on medications for chronic diseases which inevitably required them to visit the pharmacy more frequently. A review of patient satisfaction with pharmacy services showed that the higher the frequency of counselling and monitoring, the greater the satisfaction rating [28]. However, another study found that seeing a healthcare provider for an old problem was associated with a low level of satisfaction [29]. It indicated that the patients with old problems had the odds of 0.51 and 0.27 for satisfaction for provider and quality of care, respectively. Meanwhile, researchers in Nigeria found no significant association between the number of visits to the pharmacy with patient satisfaction towards pharmacy services [12].

This current study showed that higher self-perceived health status score was significantly associated with higher patient satisfaction score which was similar to previous studies [16, 17, 21, 29]. A study by Xiao & Barber (2008) indicated that patients with good to excellent self-perceived physical health status had higher odds for satisfaction with access to care, provider and quality of care (OR: 2.03, OR: 1.49 and OR: 1.43, respectively) [29]. As patient satisfaction is influenced by the patient’s well-being, patients who perceive themselves in better health status may associate their sense of well-being with the care received and report higher level of satisfaction [29]. Our findings also showed a significant interaction between age and self-perceived health status with patient satisfaction. This was similar to a study by Jackson et al. (2001) which found that older patients who perceived themselves in better health status would express higher level of patient satisfaction [27]. Older patients tend to perceive themselves in poorer health status due to deteriorating health conditions, hence lowering their level of satisfaction [24].

Level of education was also found to have significant interaction with self-perceived health status towards patient satisfaction where patients with higher education who perceived themselves in better health status expressed a higher level of satisfaction. A study based on the Canadian Community Health Survey of over 20,000 adults found that more educated patients tend to report better health [30]. As healthier people are less likely to need as much medical care and interact with healthcare providers less frequently, they may have less opportunity to experience problems with access to health care and express more satisfaction with the care received [29].

This study indicated that higher-level general knowledge of pharmacists among patients was significantly associated with higher levels of satisfaction towards pharmacy services. As pharmacy services evolve from traditional dispensing roles to more active patient-centred pharmaceutical care, it is essential to measure a patient's general knowledge of pharmacists. This reflects how patients perceive the expertise of pharmacists and expectations of pharmacy services, which in turn affects the level of patient satisfaction [31, 32]. AlGhurair et. al. (2012) also observed perceived expertise was an independent determinant of patient satisfaction [31]. They reported a significant, positive correlation between perceived expertise and patient satisfaction (*r* = 0.52; *p* = 0.001).

Our study had few strengths including a newly developed questionnaire to measure patient satisfaction specifically towards patients attending public health clinics and adding information on the patient’s general knowledge of pharmacists in relation to patient satisfaction as local studies on this issue are limited. Our study presented some limitations. Approximately 70% of items could be interpreted as possessing a ceiling effect where the mean score for patient satisfaction was >8.00 for 16 out of 22 items. Although this may reflect patients' positive evaluation of the care received, acquiescence bias needs to be considered as patients may be reluctant to express critical comments or concerns and inclined to accept their care unless something untoward happens [4]. The fact that patients were required to recall the number of visits to the pharmacy may have attributed to recall bias.

## Conclusions

A new tool was developed to measure patient satisfaction towards pharmacy services in public health clinics in Malaysia. Patients expressed a relatively high level of satisfaction towards pharmacy services. Special attention should be given towards the elderly, patients with high education, first-time visitors to the pharmacy, patients who perceive themselves in poorer health status and those who have poor general knowledge of pharmacists.

## Supporting information

S1 Appendix Public Health Clinic-Patient Satisfaction Questionnaire (PHC-PSQ). (PDF)
