# Supplementary material for: Factors associated with patient satisfaction towards pharmacy services among out-patients attending public health clinics: Questionnaire development and its application
Source: PLoS One. 2020 Nov 10;15(11):e0241082. doi: 10.1371/journal.pone.0241082 (PMC7654767; doi:10.1371/journal.pone.0241082)
Supplement: S1 Appendix — (PDF) [file pone.0241082.s001.pdf]

Supporting Information

1. Questionnaire tool – Public Health Care Patient Satisfaction Questionnaire (PHC-PSQ)

Code Number: ☐

Public Health Care Patient Satisfaction Questionnaire (PHC-PSQ)

Please fill in the form and tick (√) in the appropriate box.

Section A: Patient profile

1) Age: _________ years old

2) Gender: Male ☐
Female ☐

3) Race: Malay ☐ Others (please state): ______________
Chinese ☐
Indian ☐

4) Level of Education: Primary school ☐
Secondary school ☐
College ☐
University ☐

5) Employment Status: Government servant ☐ Unemployed ☐
Private employee ☐ Housewife ☐
Self-employed ☐ Student ☐
Retiree ☐

6) Monthly Income: RM _______________ None ☐

Section B: Frequency of visit to pharmacy

In the past 3 months, how many times have you visited this pharmacy?

1 ☐ 2 ☐ 3 ☐ If more than 3 times (please state): ______

Section C: Self-perceived health status

Please tick (√) in the appropriate box.	Very poor 1	2	3	4	Very good 5
Please rate your current health status.	☐	☐	☐	☐	☐

Section D: General knowledge of pharmacists

No.	Please tick (√) in the appropriate box.	True	False	Not Sure
What do you think of the following statements?				
1	Pharmacists are healthcare professionals.			
2	Every pharmacist must have university degree qualification.			
3	Pharmacists need to complete one (1) year of supervised training after graduation.			
4	Pharmacists do not need to register with the Pharmacy Board of Malaysia prior to working in a pharmacy.			
5	Pharmacists can dispense high blood pressure medications without prescription from a doctor.			
6	Pharmacists can provide drug information to patients and other healthcare professionals.			
7	Pharmacists know how to identify side effects of medications and drug interactions.			
8	Pharmacists can advise on how to manage minor illnesses.			
9	Pharmacists can assist in monitoring of chronic diseases and advise patients to see a doctor when necessary.			
10	Pharmacists can prescribe antibiotics.			

Section E: <u>Patient satisfaction towards</u> <u>pharmacy service in this clinic</u>

No.	Please rate the following items and circle the number in the appropriate box.	Not satisfied 1	2	3	4	5	6	7	8	9	Very satisfied 10
**a)**	**Administrative Competency**										
1	Directions to the pharmacy are clear.	1	2	3	4	5	6	7	8	9	10
2	The pharmacy is well maintained.	1	2	3	4	5	6	7	8	9	10
3	The pharmacy is clean.	1	2	3	4	5	6	7	8	9	10
4	The waiting area is comfortable.	1	2	3	4	5	6	7	8	9	10
5	There is sufficient seating in the waiting area.	1	2	3	4	5	6	7	8	9	10
6	The pharmacy counter is comfortable.	1	2	3	4	5	6	7	8	9	10
7	Operating hours of the pharmacy are satisfactory.	1	2	3	4	5	6	7	8	9	10
8	The waiting time to get a **<u>queue number</u>** at the pharmacy counter is short.	1	2	3	4	5	6	7	8	9	10
9	The waiting time to get my **<u>medication</u>** at the pharmacy counter is short.	1	2	3	4	5	6	7	8	9	10
**b)**	**Technical Competency**										
1	The pharmacist is polite and friendly.	1	2	3	4	5	6	7	8	9	10
2	The pharmacist provides medication with clear drug label and explanation.	1	2	3	4	5	6	7	8	9	10
3	The pharmacist listens to what I have to say.	1	2	3	4	5	6	7	8	9	10
4	The pharmacist explains how to take the medications and why it is important to take my medications as directed.	1	2	3	4	5	6	7	8	9	10
5	The pharmacist always explains the side effects of medications.	1	2	3	4	5	6	7	8	9	10
6	The pharmacist ensures I fully understand the explanation given.	1	2	3	4	5	6	7	8	9	10
7	The pharmacist is helpful when I have problems with my medications.	1	2	3	4	5	6	7	8	9	10
8	The pharmacist ensures the medication I need is always in stock.	1	2	3	4	5	6	7	8	9	10
9	The pharmacist provides sufficient health-related reading materials such as posters and leaflets.	1	2	3	4	5	6	7	8	9	10
10	I feel confident that the drug information provided by the pharmacist is accurate.	1	2	3	4	5	6	7	8	9	10

No.	Please rate the following items and circle the number in the appropriate box.	Not satisfied 1	2	3	4	5	6	7	8	9	Very satisfied 10
**c)**	**Convenient Location**										
1	There are sufficient parking spaces close to the pharmacy.	1	2	3	4	5	6	7	8	9	10
2	The pharmacy is near public transport.	1	2	3	4	5	6	7	8	9	10
3	The pharmacy is near my home.	1	2	3	4	5	6	7	8	9	10

Thank you for your participation in this survey!

Your contribution will help improve the quality of pharmacy services!

Please return the completed questionnaire to the pharmacy.
